# Supplementary material for: Chiral orbital lasing in a twisted bilayer metasurface
Source: Nat Commun. 2026 Mar 12;17:2369. doi: 10.1038/s41467-026-69665-w (PMC12982590; doi:10.1038/s41467-026-69665-w)
Supplement: Supplementary file 1 — Supplementary Information [file 41467_2026_69665_MOESM1_ESM.pdf]

Supplementary Information — Chiral orbital lasing in a twisted bilayer metasurface

Mingjin Wang[1,2,3,4,#], Nianyuan Lv[5,#], Zixuan Zhang[5,#], Ye Chen[5], Jiahao Si[1], Jingxuan Chen[1], Chenyan Tang[1], Xuefan Yin[5], Zhen Liu[5], Dongxu Xin[1], Zhaozheng Yi[1], Wanhua Zheng[1,2,3,4,*], Yuri Kivshar[6,7,*], and Chao Peng[5,8,*]

[1]Laboratory of Solid State Optoelectronics Information Technology, Institute of Semiconductors, CAS, Beijing 100083, China

[2]Center of Materials Science and Optoelectronics Engineering, University of Chinese Academy of Sciences, Beijing 100049, China

[3]Hangzhou Institute for Advanced Study, University of Chinese Academy of Sciences, Hangzhou 310024, China

[4]College of Future Technology, University of Chinese Academy of Sciences, Beijing 101408, China

[5]State Key Laboratory of Photonics and Communications, School of Electronics & Frontiers Science Center for Nano-optoelectronics, Peking University, Beijing 100871, China

[6]Nonlinear Physics Centre, Research School of Physics, Australian National University, Canberra ACT 2601, Australia

[7]Department of Physics, The University of Hong Kong, Hong Kong 999077, China

[8]Peng Cheng Laboratory, Shenzhen 518055, China

[1]These authors contributed equally to this work.

[*]To whom correspondence should be addressed: Wanhua Zheng (E-mail: whzheng@semi.ac.cn), Yuri Kivshar (E-mail: yuri.kivshar@anu.edu.au), Chao Peng (E-mail: pengchao@pku.edu.cn).

Supplementary Note 1. Detailed material, structure design and fabrication

We begin by detailing the material composition and structural design of our InP-based twisted bilayer metasurface system. Notably, our structure features two symmetric membrane sheets made from InGaAsP multiple quantum wells (MQWs) suspended in air, with each sheet patterned by square-lattice periodic air holes defined by the lattice constant a, hole radius r, and membrane thickness h. The two sheets rotate and overlay as a twisted bilayer system, depicted by twist angle θ and gap distance g.

Each suspended membrane is composed of an upper cladding layer (246.5 nm InGaAsP), an active region (129 nm in total), and a lower cladding layer (246.5 nm InGaAsP). The active region consists of six compressively strained InGaAsP quantum wells (7.5 nm thick each) and seven lattice-matched, tensile-strained barriers (12 nm thick each). Beneath the MQWs lies a 1000 nm InP sacrificial layer, which is removed via wet etching to suspend the structure. The air holes are etched completely through each slab, maintaining z-axis symmetry in both layers. We design the metasurface with 50×50 air holes at a lattice constant $a = 540$ nm with the depth and radius of the circular hole being $h = 622$ nm and $r = 153$ nm, respectively. The gap distance between the upper and lower sheets is designed as $g = 100$ nm. As mentioned in the main text, the twist generates a Moiré pattern, leading to the formation of a supercell with a real-space size $L = Na$ (Fig. 1d). The twist angle θ determines the size of the supercell as $N = 1/(\sqrt{2}sin(\theta/2))$. At a twisted angle of 22.62° in our design, the supercell size of the Moiré pattern is $5/\sqrt{2}a \times 5/\sqrt{2}a$.

The sample fabrication relies on electron-beam lithography (EBL) for precise alignment of metasurface membranes and high-aspect-ratio inductively coupled plasma (ICP) etching for

the InP-based epi-wafer. A silicon dioxide hard mask is first deposited on the substrate via plasma-enhanced chemical vapor deposition (PECVD), prior to spin-coating a 500 nm thick layer of Zep-520A photoresist at 4000 rpm. Following a softbake, the epi-wafers are exposed by EBL (ELX-F125) under the following parameters: beam energy of 125 keV, beam spot size of 2 nm, and exposure dose of 380 μC/cm^2. After developing with fresh developer and undergoing a series of cleaning processes, the silicon dioxide hard mask is fabricated via ICP dry etching. Selective dry etching of silicon dioxide is performed for 250 s under an inductive coupling power of 900 W, an RF bias power of 50 W, a chamber pressure of 3.75 mTorr, and gas flow rates of 10/30 sccm for CHF_3/CF_4. Subsequently, the metasurface on the InP epi-wafers is fabricated using a PlasmaPro 100 Cobra system (Oxford Instruments). A fast etch rate is achieved in 60 s under an inductive coupling power of 500 W, an RF bias power of 170 W, a chamber pressure of 5 mTorr, gas flow rates of 12/2/30 sccm for BCl_3/Cl_2/Ar, and a temperature of 220 °C.

Supplementary Note 2. Quadratic and isotropic dispersion of Moiré supercell

When twisted metasurfaces form a Moiré lattice, the interlayer coupling alters the band dispersions. As outlined in the main text, our investigation focuses on the TE-A band near the Γ point, which exhibits fundamentally different behaviors from the reported flat-band effect in lasing, at which the coupling flattens the bands in the vicinity of the K point [1, 2]. In our design, we employ a moderately sized supercell ($5/\sqrt{2}a \times 5/\sqrt{2}a$) with a relatively large twist angle of $\theta = 22.62°$. Numerical simulations (COMSOL Multiphysics) reveal that the TE-A band in the individual layer hybridizes into two distinct modes with opposite z-symmetry, referred to as the odd and even bands (Supplementary Fig. 1a). We further analyze the band dispersion near the BZ center of the Moiré supercell, which remains notably quadratic rather than flat. Additionally, the dispersion is found to be highly isotropic in reciprocal space, as evidenced by the nearly circular iso-frequency contours as shown in Supplementary Fig. 1b.

The quadratic and isotropic dispersion of our Moiré supercell gives rise to a unique collective oscillation behavior [3]. As shown in Supplementary Fig. 1c, unlike the flat-band effect [4, 5, 6, 7], the hybridized mode extends across the entire supercell rather than being confined to a few unit-cells. This delocalization occurs because the group velocity is nonzero upon the quadratic band. As a result, light can propagate through multiple supercells as “bulk guided resonances," which can further support collective guided resonances (CGRs) when confined within an effective cavity region. Second, the isotropic band dispersion reflects rotational symmetry at any angle. Combined with the circular shape of the effective cavity, the resulting CGRs modes also possess continuous rotational symmetry, rotating in either clockwise (CW) or counterclockwise (CCW) directions, respectively, showing as twofold degeneracy in a single

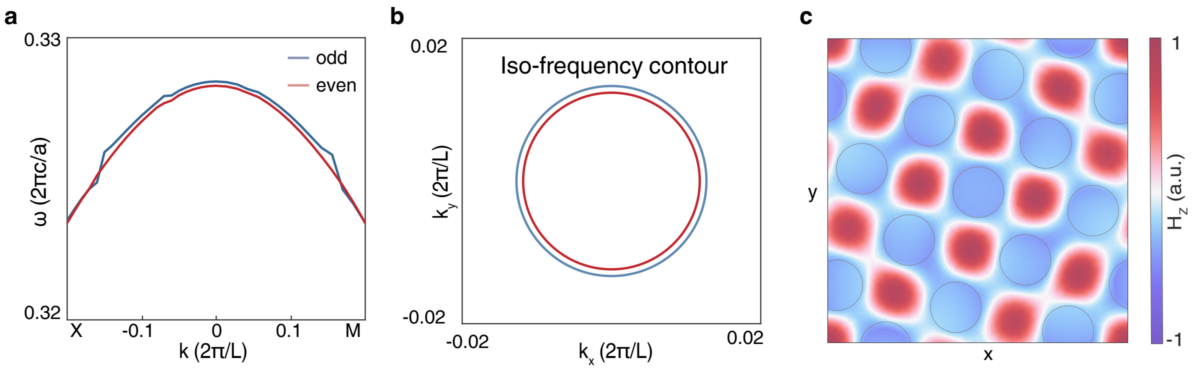

Supplementary Figure 1: **The dispersion of our Moiré supercell.** (a) The band structure near the BZ center of the supercell with quadratic dispersions. (b) The corresponding iso-frequency contours, showing the dispersions are isotropic. (c) The real space H_z distribution at the supercell's BZ center.

layer.

To rigorously validate the origin of the Bloch modes, we further performed full-wave eigenfrequency simulations on the complete twisted bilayer supercell. Supplementary Figure 2 displays the calculated band structure, which reveals a complex spectral landscape typical of Moiré lattices. Due to the reduction of the Brillouin zone into a mini-Brillouin zone, the original photonic bands undergo extensive zone folding, resulting in a dense set of bands. The light cone is clearly demarcated by the dashed lines.

Despite the spectral complexity induced by the zone folding, the modes of primary interest remain clearly identifiable. As shown in the zoomed-in view in Supplementary Fig. 3 (corresponding to the red box in Supplementary Fig. 2), the interlayer coupling lifts the degeneracy of the folded bands at the Γ point. The hybridized odd and even states originating from

the fundamental TE-A band are distinctly isolated from other folded bands in the frequency vicinity. This isolation confirms that, within the high Q region relevant to lasing, the complex supercell physics can be accurately captured by the effective model focusing on these specific quadratic branches.

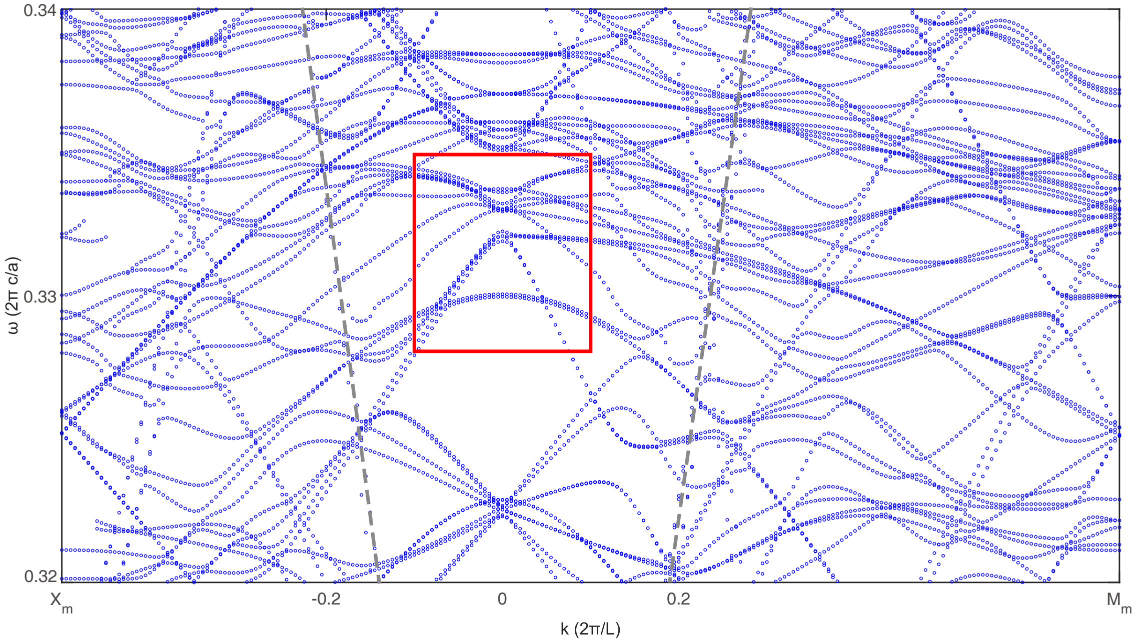

Supplementary Figure 2: **Full band structure of the twisted bilayer Moiré supercell.** Calculated eigenfrequency dispersion along the high-symmetry directions of the mini-Brillouin zone. The spectrum exhibits a dense set of bands arising from zone-folding due to the large real-space periodicity. The red box indicates the frequency range of interest.

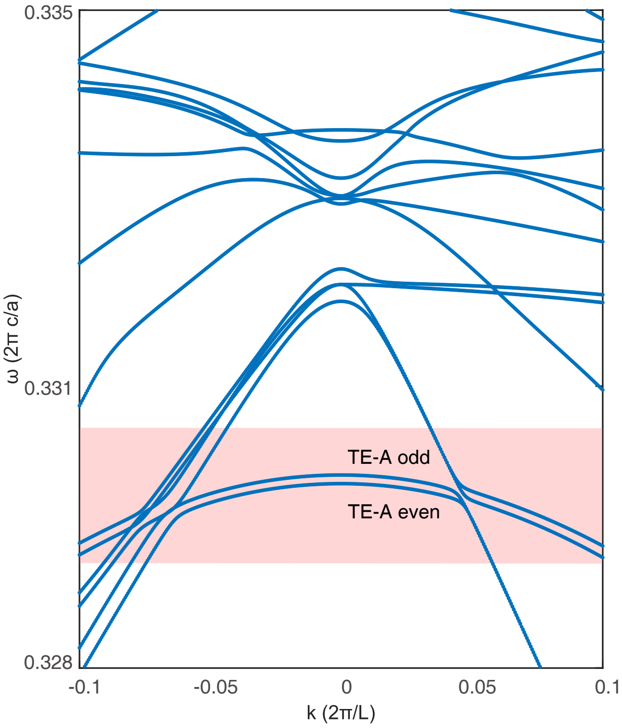

Supplementary Figure 3: **Zoomed-in view of the region of interest.** A Zoomed-in view of the region highlighted in Supplementary Fig. 2. The hybridized TE-A odd and even bands (candidate lasing modes) are clearly identified and remain relatively isolated from other folded bands, validating the applicability of the effective Hamiltonian model.

Supplementary Note 3. Radiation suppression and air gap optimization in Moiré lattice

To support lasing oscillation, optical modes must achieve a sufficiently high Q. Unlike reported Moiré lattice cases where modes reside at the K point [1, 2], our mode operates at the Γ point. As a result, it naturally couples to free space and tends to radiate energy. However, this radiation loss can be suppressed through the strategic arrangement of bound states in the continuum (BICs). The simplest way is to utilize the symmetry-protected BIC at the Γ point. By decreasing the twist angle, it was reported that the Moiré mode at the BZ center gradually evolves into a near-perfect BIC, whose radiation losses are significantly reduced [8]. However, the twist angle in our design needs to be relatively large to support quadratic dispersion. In this case, the radiation loss can be almost eliminated by aligning multiple topological polarization charges across all diffraction channels, to enable robust radiation suppression across the entire Moiré band. In physics, diffraction from Moiré lattices generates multiple diffracted waves, some of which fall within the light cone (Supplementary Fig. 4a). These components lead to out-of-plane energy leakage, thereby reducing the Qs of the optical modes. To suppress this radiation loss, we adopt a two-step approach in designing the geometric parameters as follows:

First, we chose a relatively large twist angle to shrink the supercell's size. Accordingly, only a few diffracted orders were left in the light cone. At $\theta = 22.62°$ with $N = 5$ that compromises the quadratic and isotropic dispersion requirement mentioned above, only four diffracted orders remain radiative that are residing at the X and Y directions in the momentum space, showing as light bright spots in the far-field (Supplementary Fig. 4b). Second, we apply tunable BICs to further suppress the radiation. Specifically, we fine-tune the slab thickness to $h = 1.21a$ (Supplementary Fig. 4c). The BICs that carry integer topological charges evolve along the X and Y directions in the momentum space until they coincide with the diffraction

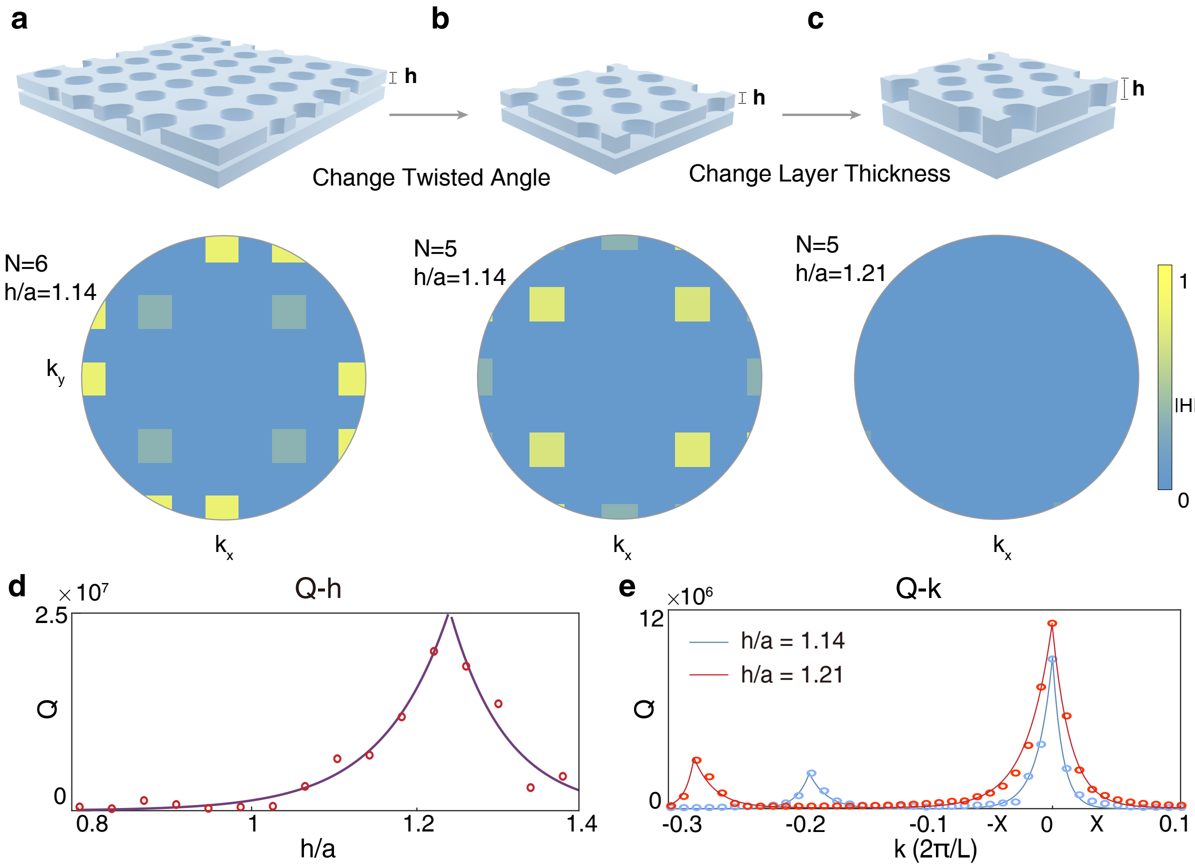

Supplementary Figure 4: **Radiation suppression of Moiré lattice.** (a) For a Moiré supercell $N = 6$ and membrane thickness $h = 1.14a$ (upper panel), the radiation pattern in reciprocal space (lower panel) include multiple diffraction orders. (b) By shrinking the supercell size to $N = 5$ while keeping $h = 1.14a$ (upper panel), only a few diffraction orders remain but are still leaky (lower panel). (c) By increasing the thickness to $h = 1.21a$ while keeping $N = 5$ (upper panel), the arrangement of BICs suppresses most radiation, evidenced by a darker radiation pattern. (d) The TE-A mode's Q in a single metasurface on varying the thickness h, showing the optimal Q reaches 2.5×10^7. (e) The Qs of Moiré supercell modes in the reciprocal space, indicating the optimal design is achieved when the BICs match with the diffracted momentum.

orders. Combined with the symmetry-protected BIC at the BZ center, the residual radiations are effectively suppressed. As shown in the lower panel of Supplementary Fig. 4c, the entire light cone becomes considerably dark.

We verified our design via numerical simulations (COMSOL Multiphysics), indicating that the Q at the supercell BZ center reaches 2.5×10^7 at $h = 1.21a$ (Supplementary Fig. 4d), which is high enough to support the lasing action. The Qs of the TE-A band in the Moiré supercell are plotted in Supplementary Fig. 4e for two different slab thicknesses, clearly showing the effectiveness of the BIC arrangement.

While the membrane thickness is optimized to suppress radiation channels via BIC alignment, the interlayer air gap constitutes another critical degree of freedom that determines the lasing performance. The selection of the air gap size involves a strategic trade-off between the interlayer coupling strength (manifested as mode frequency splitting) and the cavity Q. We performed numerical simulations to map the evolution of the supercell eigenmodes as a function of the air gap thickness. As illustrated in Supplementary Fig. 5a, the interlayer coupling strength decays as the air gap increases, causing the eigenfrequencies of the TE-A odd and even modes to converge. If the gap is too large, the modes become spectrally indistinguishable, which hinders the isolation of the desired chiral mode. Conversely, reducing the air gap enhances coupling but reduces the Q factor (as shown in Supplementary Fig. 5b), which is detrimental to low-threshold lasing. Furthermore, an extremely narrow air gap imposes significant fabrication challenges; specifically, reliable deposition processes typically require a minimum thickness of approximately 50 nm to ensure the structural flatness and quality of the spacing layer. Consequently, we selected an optimal air gap of 100 nm to maintain sufficient mode splitting for spectral isolation while preserving a high Q factor to support efficient lasing oscillation.

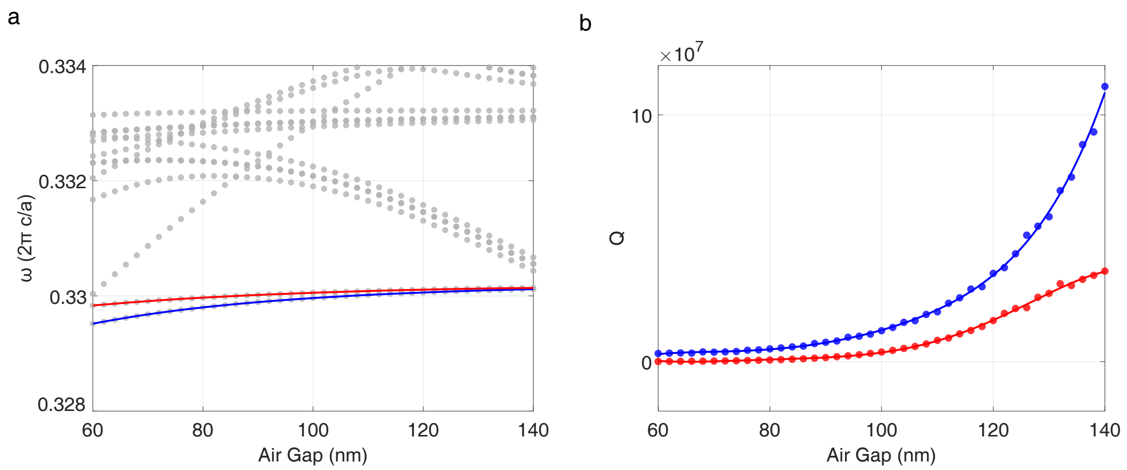

Supplementary Figure 5: **Optimization of the interlayer air gap.** (a) The eigenfrequencies for the TE-A odd (red) and even (blue) modes as a function of the air gap thickness. A larger gap reduces the frequency splitting, indicating weaker interlayer coupling. (b) Q factor for the corresponding modes. The air gap of 100 nm is chosen as a trade-off to ensure distinguishable mode splitting and sufficiently high Q for lasing, while satisfying fabrication constraints.

Supplementary Note 4. Laguerre-Gaussion modes in a round effective cavity

As discussed in the main text, the combination of isotropic dispersion and a rotational invariant cavity leads to twofold degenerate modes. In this section, we analytically solve the wave equation in polar coordinates and demonstrate that such modes can be described by Laguerre-Gaussian functions when a round gain-guided effective cavity is introduced.

When the twisted metasurface on the MQWs wafer is optically pumped, carrier diffusion results in a spatially non-uniform gain profile, which in turn modifies the refractive index. As an example, we consider a circular pump beam, which produces an isotropic refractive index distribution as:

$$n(\rho) = n_1(0) - n_2\rho^2 \tag{S1}$$

where ρ is the radial position, n_1 and n_2 are coefficients dependent on the material and pump power. Furthermore, due to the finite size of the incident pump spot, the variation in carrier density (and thus the change in refractive index) in the unpumped regions can be neglected. As a result, the pump spot effectively imposes a circular boundary condition on the optical mode under gain-guided operation. Substituting Eq.S1 into Maxwell's equations

$$[\frac{\partial^2}{\partial\rho^2} + \frac{1}{\rho}\frac{\partial}{\partial\rho} + \frac{1}{\rho^2}\frac{\partial^2}{\partial\phi^2} + k_0^2(\xi_0(z) - \bar{a}^2\rho^2)](\psi\rho) = \beta_0^2\psi(\rho) \tag{S2}$$

where ϕ is the azimuth angle; $\bar{a} = \sqrt{2n_1(0)n_2}/k_0 = a_r + ia_i$ is determined by the material properties. With such a refractive index profile, the finite-size modes follow slowly varying envelope approximation, in forms of Laguerre-Gaussian functions[9]:

$$\psi_{pl}(\rho,\phi) = \rho_n^{|l|}\exp(-\rho_n^2/2)L_p^{|l|}(\rho_n^2)\exp(il\phi) \tag{S3}$$

where $\rho_n = \sqrt{k_0\bar{a}}\rho$ is a dimensionless radial position, and $L_p^{|l|}(\rho^2)$ represents the generalized Laguerre polynomial with radial index p and azimuthal index l. Further, the corresponding

eigenvalues are:

$$\beta_0^2 = k_0^2\xi_0(z) - 2k_0\bar{a}(2p + |l| + 1) \tag{S4}$$

By comparing with the standard expression of Laguerre-Gaussian vortex beams carrying the orbital angular momentum (OAM)[10, 11]:

$$\psi_{pl}(r, \phi) = C\left(\frac{\sqrt{2}r}{w_0}\right)^{|l|} L_p^{|l|}\left(\frac{2r^2}{w_0^2}\right)\exp\left(-\frac{r^2}{w_0^2}\right)\exp\left(-il\phi\right) \tag{S5}$$

where C is a normalized constant and w_0 is the beam waist radius, we notice our solution of Eq. S3 is exactly the Laguerre-Gaussian function. Therefore, the gain-guided effective cavity indeed enables in-plane confinement, and minimizes scattering at the outer boundaries of the cavity.

The above result stems from the isotropic dispersion and geometry of our twisted metasurface system. In our device, both the gain profile, boundary conditions, and band dispersions are isotropic, obeying rotational symmetry at an arbitrary angle. Recall the fact that the bulk guided resonances that participate in the collective hybridization need to align with the isofrequency contour in momentum space. Under the omnidirectional scattering enforced by the circular boundary conditions, each component contributes with equal weighting, thus creating Laguerre-Gaussian profiles as we solved.

Supplementary Note 5. Helical and non-Hermitian couplings in twisted bilayer metasurface

The twisted metasurface's inherent chirality gives rise to a specific coupling scenario. As mentioned in the main text, we consider Laguerre-Gaussian functions $|\psi^{u,\,l}_{CW,\,CCW}\rangle$ in the upper and lower sheets as the unperturbed basis, which can rotate in CW or CCW directions. The coupling paths can be categorized into three distinct types, namely intra-layer cross-coupling ($\kappa^{\text{intra}}_{1,2}$), inter-layer cross-coupling ($\kappa^{\text{inter}}_{1,2}$), and inter-layer direct-coupling ($\kappa^{\text{inter}}_{\text{direct}}$). Here, the terms "cross" and "direct" refer to the couplings occurring between modes with opposite or identical rotating directions, respectively. We note that all these couplings are intrinsically non-Hermitian due to the presence of radiation and material gain/loss, while the cross couplings are helical owing to the chiral nature of the twisted metasurface.

We first discuss the intralayer cross-coupling effect. As schematically illustrated in Supplementary Fig. 6, we consider the modes in the lower metasurface, namely $|\psi^{\,l}_{CW}\rangle$ and $|\psi^{\,l}_{CCW}\rangle$. In this case, the upper one can be regarded as an asymmetric scatterer in real space. Seeing from the lower one, the upper metasurface breaks its mirror symmetry, meaning the system cannot be mapped onto its mirror image, so the coupling becomes directional to the helicity. Moreover, the coupling process involves radiation, absorption, and gain, rendering the coupling inherently non-Hermitian, revealing that the coupling coefficients are complex numbers. Specifically, for the lower metasurface, we describe the presence of the upper one as a perturbed Hamiltonian $\Delta\mathbf{H}$. The intralayer coupling between CW and CCW modes in the lower metasurface can be expressed as:

$$\kappa_1^{\text{intra}} = \kappa^{\text{intra}}_{l,CW\rightarrow CCW} = \langle\psi^{l}_{CCW}|\Delta H|\psi^{l}_{CW}\rangle = \int_{upper} \psi^{*,l}_{CCW}(z)\psi^{l}_{CW}(z)\Delta H(z)dz \tag{S6}$$

Here, the integral is applied to the upper membrane region where the asymmetric scatter resides,

and $\psi^{*,l}_{CCW}(z)\psi^{l}_{CW}(z)$ presents the overlap in such a region. Since the $\Delta\mathbf{H}$ is non-Hermitian and helical, we got $\kappa_1^{\text{intra}} = \kappa^{\text{intra}}_{l,CW\to CCW} \neq \kappa^{\text{intra}}_{l,CCW\to CW} = \kappa_2^{\text{intra}}$.

A similar argument holds for interlayer cross-coupling. We consider the CW mode in the upper metasurface $|\psi^{u}_{CW}\rangle$ coupled with the CCW mode in the lower one $|\psi^{l}_{CCW}\rangle$ (Fig. S6), and we treat one sheet as an asymmetric scatterer to the other. The coupling coefficient can be written as:

$$\begin{aligned}\kappa_1^{\text{inter}} &= \kappa^{\text{inter}}_{l,CW\to CCW} + \kappa^{\text{inter}}_{u,CW\to CCW} = \langle\psi^{l}_{CCW}|\Delta H|\psi^{u}_{CW}\rangle + \langle\psi^{u}_{CCW}|\Delta H|\psi^{l}_{CW}\rangle \\ &= \int_{upper}\psi^{*,l}_{CCW}(z)\psi^{u}_{CW}(z)\Delta H(z)dz + \int_{lower}\psi^{*,u}_{CCW}(z)\psi^{l}_{CW}(z)\Delta H(z)dz \end{aligned} \tag{S7}$$

which shows that the coupling simultaneously happens at both upper and lower sheets, creating helical and non-Hermitian coupling coefficient as $\kappa_1^{\text{inter}} = \kappa^{\text{inter}}_{u+l,CW\to CCW} \neq \kappa^{\text{inter}}_{u+l,CCW\to CW} = \kappa_2^{\text{inter}}$.

The third coupling path is the direct coupling between different layers while the mode has the same helicity. We take the CW mode for example, the coupling coefficient is given by:

$$\kappa^{\text{inter}}_{\text{direct}} = \kappa^{\text{inter}}_{CW\to CW} = \langle\psi^{u}_{CW}|\Delta H|\psi^{l}_{CW}\rangle = \int_{entire}\psi^{*,u}_{CW}(z)\psi^{l}_{CW}(z)\Delta H(z)dz \tag{S8}$$

Because the compound twisted bilayer system has the same average permittivity under mirror operation, we got $\kappa^{\text{inter}}_{\text{direct}} = \kappa^{\text{inter}}_{CW\to CW} = \kappa^{\text{inter}}_{CCW\to CCW}$, showing the direct couplings are not helical but non-Hermitian.

The above discussion reveals that our twisted bilayer metasurface system is intrinsically chiral and non-Hermitian. This leads to more complex dynamics, including asymmetric coupling rates between modes and the spawn of exceptional points for chiral lasing as elaborated in the following section.

While the discussion above relies on symmetry arguments to construct the Hamiltonian, the coupling coefficients κ are physical quantities that can be rigorously derived using electromagnetic perturbation theory. The effective Hamiltonian describes the interaction between mode i (with electric field $\mathbf{E}_i$) and mode j (with electric field $\mathbf{E}_j$) mediated by a dielectric perturbation $\Delta\epsilon$. The generic form of the coupling coefficient κ_{ij} is given by the overlap integral.

$$\kappa_{ij} \approx \frac{\omega_0}{2} \frac{\int_V \Delta\epsilon(\mathbf{r})\mathbf{E}_i^*(\mathbf{r}) \cdot \mathbf{E}_j(\mathbf{r}) dV}{\int_V \epsilon(\mathbf{r})|\mathbf{E}_i(\mathbf{r})|^2 dV} \tag{S9}$$

Based on this formulation, the specific coupling mechanisms in our twisted bilayer system can be physically defined.

Intra-layer Cross-Coupling (κ^{intra}): For the intra-layer coupling (e.g., coupling between CW and CCW modes confined in the lower layer), the perturbation $\Delta\epsilon$ arises from the dielectric grating of the adjacent upper metasurface. Since the upper metasurface is rotated by a twist angle θ, this perturbation introduces the necessary momentum kick. The coupling strength is proportional to the overlap within the volume of the perturbing layer:

$$\kappa_{1,2}^{\text{intra}} \propto \int_{V_{\text{upper}}} \Delta\epsilon_{\text{upper}}(\mathbf{r}, \theta)\mathbf{E}_{\text{CCW}}^{*,l}(\mathbf{r}) \cdot \mathbf{E}_{\text{CW}}^{l}(\mathbf{r}) dV \tag{S10}$$

Inter-layer Coupling (κ^{inter}): Similarly, the inter-layer coupling is dictated by the overlap of the evanescent tails of the modes in the gap region and the material of the opposite layer. For

instance, the direct coupling strength is determined by:

$$\kappa_{\text{direct}}^{\text{inter}} \propto \int_V \Delta\epsilon_{\text{gap}}(\mathbf{r})|\mathbf{E}_{\text{CW}}(\mathbf{r})|^2 dV \tag{S11}$$

The non-Hermitian coupling coefficients can also be derived, in which $\Delta\epsilon$ is introduced by the adjacent layer, as

$$\kappa_1^{\text{inter}} = \frac{\omega_0}{2} \int \mathbf{E}_{CCW}^{*,u}(\mathbf{r}) \cdot \Delta\epsilon(\mathbf{r}) \cdot \mathbf{E}_{CW}^{l}(\mathbf{r})\, dV \tag{S12}$$

Numerical Extraction of Coefficients: Although the coupling coefficients are calculable in principle via the integrals above, a full-wave simulation of the entire twisted supercell system required to retrieve accurate field distributions is computationally prohibitive due to the large Moiré scale. Consequently, we extract the coupling values used in our model from the spectral splitting observed in numerical eigenmode simulations of the supercell.

First, the splitting between the even and odd supermodes is dominated by the direct interlayer coupling $\kappa_{\text{direct}}^{\text{inter}}$. The observed normalized frequency difference of $\Delta(a/\lambda) \approx 9 \times 10^{-5}$ (presented in Supplementary Fig. 2 and 3) corresponds to a direct coupling strength of $\kappa_{\text{direct}}^{\text{inter}} \approx 2.6 \times 10^{-4}$ in normalized units. Second, the fine spectral splitting observed prior to lasing (approximately 0.23 nm) reflects the interplay between inter- and intra-layer chiral couplings. This experimentally and numerically consistent splitting corresponds to a chiral coupling strength on the order of $\kappa^{\text{intra}} \approx 10^{-4}$. These extracted values ensure that our effective Hamiltonian quantitatively reproduces the spectral features of the twisted bilayer system.

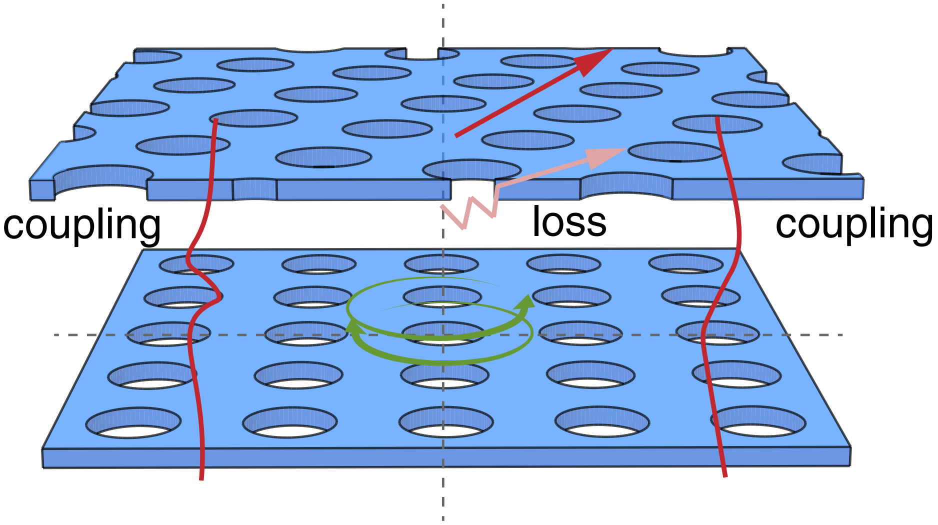

Supplementary Figure 6: **Helical and non-Hermitian coupling processes in the twisted bilayer metasurfaces.** For instance, from the perspective of the lower metasurface, the upper one breaks its mirror symmetry, which makes the coupling process helical. Besides, the system possesses out-of-plane radiation and material gain/loss, so the coupling processes are generally non-Hermitian.

Supplementary Note 6. The emergence of exception points and intrinsic orbital chirality

According to the coupling paths and their strengths derived above, we write an effective Hamiltonian to depict the interplay of the four Laguerre-Gaussian functions in the upper and lower metasurfaces as the basis [12], as $\mathbf{V} = [|\psi^{u}_{CCW}\rangle,\ |\psi^{u}_{CW}\rangle,\ |\psi^{l}_{CCW}\rangle,\ |\psi^{l}_{CW}\rangle]^T$, which is a 4×4 coupling matrix corresponding to the following eigenvalue problem:

$$\mathbf{H}_{\text{eff}}\mathbf{V} = \begin{pmatrix} \Omega_0 & \kappa_1^{\text{intra}} & \kappa_{\text{direct}}^{\text{inter}} & \kappa_1^{\text{inter}} \\ \kappa_2^{\text{intra}} & \Omega_0 & \kappa_2^{\text{inter}} & \kappa_{\text{direct}}^{\text{inter}} \\ \kappa_{\text{direct}}^{\text{inter}} & \kappa_1^{\text{inter}} & \Omega_0 & \kappa_1^{\text{intra}} \\ \kappa_2^{\text{inter}} & \kappa_{\text{direct}}^{\text{inter}} & \kappa_2^{\text{intra}} & \Omega_0 \end{pmatrix}\mathbf{V} = \Omega'\mathbf{V} \tag{S13}$$

Here, Ω_0 is the unperturbed frequency of $|\psi^{u,\,l}_{CW,\,CCW}\rangle$; Ω' is the eigenvalue of the hybridized states through the couplings. The coupling coefficient configuration is consistent with the system's symmetry, where the upper and lower metasurfaces are identical but twisted relative to each other by a twist angle θ. By solving this eigenproblem, we get four eigenvalues as follows:

$$\Omega_1' = \Omega_0 + \kappa_{\text{direct}}^{\text{inter}} + \sqrt{(\kappa_1^{\text{intra}} + \kappa_1^{\text{inter}})(\kappa_2^{\text{intra}} + \kappa_2^{\text{inter}})} \tag{S14}$$

$$\Omega_2' = \Omega_0 + \kappa_{\text{direct}}^{\text{inter}} - \sqrt{(\kappa_1^{\text{intra}} + \kappa_1^{\text{inter}})(\kappa_2^{\text{intra}} + \kappa_2^{\text{inter}})} \tag{S15}$$

$$\Omega_3' = \Omega_0 - \kappa_{\text{direct}}^{\text{inter}} + \sqrt{(\kappa_1^{\text{intra}} - \kappa_1^{\text{inter}})(\kappa_2^{\text{intra}} - \kappa_2^{\text{inter}})} \tag{S16}$$

$$\Omega_4' = \Omega_0 - \kappa_{\text{direct}}^{\text{inter}} - \sqrt{(\kappa_1^{\text{intra}} - \kappa_1^{\text{inter}})(\kappa_2^{\text{intra}} - \kappa_2^{\text{inter}})} \tag{S17}$$

which corresponds to eigenvectors, respectively:

$$\psi_1 = \begin{bmatrix} \sqrt{\kappa_1^{\text{intra}} + \kappa_1^{\text{inter}}}, & \sqrt{\kappa_2^{\text{intra}} + \kappa_2^{\text{inter}}}, & \sqrt{\kappa_1^{\text{intra}} + \kappa_1^{\text{inter}}}, & \sqrt{\kappa_2^{\text{intra}} + \kappa_2^{\text{inter}}} \end{bmatrix}^T \tag{S18}$$

$$\psi_2 = \begin{bmatrix} -\sqrt{\kappa_1^{\text{intra}} + \kappa_1^{\text{inter}}}, & \sqrt{\kappa_2^{\text{intra}} + \kappa_2^{\text{inter}}}, & -\sqrt{\kappa_1^{\text{intra}} + \kappa_1^{\text{inter}}}, & \sqrt{\kappa_2^{\text{intra}} + \kappa_2^{\text{inter}}} \end{bmatrix}^T \tag{S19}$$

$$\psi_3 = \begin{bmatrix} -\sqrt{\kappa_1^{\text{intra}} - \kappa_1^{\text{inter}}}, & -\sqrt{\kappa_2^{\text{intra}} - \kappa_2^{\text{inter}}}, & \sqrt{\kappa_1^{\text{intra}} - \kappa_1^{\text{inter}}}, & \sqrt{\kappa_2^{\text{intra}} - \kappa_2^{\text{inter}}} \end{bmatrix}^T \tag{S20}$$

$$\psi_4 = \begin{bmatrix} \sqrt{\kappa_1^{\text{intra}} - \kappa_1^{\text{inter}}}, & -\sqrt{\kappa_2^{\text{intra}} - \kappa_2^{\text{inter}}}, & -\sqrt{\kappa_1^{\text{intra}} - \kappa_1^{\text{inter}}}, & \sqrt{\kappa_2^{\text{intra}} - \kappa_2^{\text{inter}}} \end{bmatrix}^T \tag{S21}$$

From the analytic eigenvalues of the effective Hamiltonian, we identify that the coalescence of eigenvectors occurs under four distinct independent conditions: $\kappa_1^{\text{inter}} = \pm\kappa_1^{\text{intra}}$ or $\kappa_2^{\text{inter}} = \pm\kappa_2^{\text{intra}}$. Satisfaction of any of these conditions leads to the formation of exceptional points (EPs). Here, we focus on the specific EP condition $\kappa_1^{\text{inter}} = -\kappa_1^{\text{intra}}$ to elucidate its physical origin and consequences.

Physically, this condition is not merely a mathematical construct but signifies a critical point of destructive interference between two distinct scattering pathways induced by the twisted bilayer geometry. The coupling coefficients represent specific physical processes:

- κ^{intra} denotes the intralayer coupling, describing the scattering of a mode into its counter-propagating partner within the same layer. This process is mediated by reflections from the dielectric perturbations of the adjacent twisted metasurface.
- κ^{inter} denotes the interlayer coupling, describing the scattering of a mode into the counter-propagating partner located in the opposite layer. This process is mediated by evanescent tunneling across the vertical gap.

Consequently, the condition $\kappa_1^{\text{inter}} = -\kappa_1^{\text{intra}}$ implies that the rate of interlayer chirality trans-

fer precisely balances the rate of intralayer chirality mixing in a non-Hermitian fashion. This balance results in the destructive interference of the backward scattering channels, effectively locking the entire bilayer system into a single, unidirectional rotating state. In our experiment, this condition is accessible by tuning geometric parameters such as the interlayer gap and the twist angle.

At this specific EP, the eigenvalues and eigenvectors simplify to:

$$\Omega'_{1,2} = \Omega_0 + \kappa^{\text{inter}}_{\text{direct}} \tag{S22}$$

$$\Omega'_{3,4} = \Omega_0 - \kappa^{\text{inter}}_{\text{direct}} \pm \sqrt{2\kappa^{\text{intra}}_1(\kappa^{\text{intra}}_2 - \kappa^{\text{inter}}_2)} \tag{S23}$$

with the corresponding coalesced eigenvectors for the upper branch ($\Omega'_{1,2}$):

$$\psi_{1,2} = [0, 1, 0, 1]^T \tag{S24}$$

$$\psi_{3,4} = \left[\mp\sqrt{2\kappa^{\text{intra}}_1}, -\sqrt{\kappa^{\text{intra}}_2 - \kappa^{\text{inter}}_2}, \pm\sqrt{2\kappa^{\text{intra}}_1}, \sqrt{\kappa^{\text{intra}}_2 - \kappa^{\text{inter}}_2}\right]^T \tag{S25}$$

Such a solution reveals that the hybridized eigenmodes are divided into two branches, separated by the direct coupling strength of $\kappa^{\text{inter}}_{\text{direct}}$ as presented in Fig. 3b in the main text, while the imaginary parts of their eigen-frequencies are different due to the non-Hermitian nature of coupling. From Eq.S6-8, we found the magnitude of $\kappa^{\text{inter}}_{\text{direct}}$ is significantly larger than $\kappa^{\text{inter}}_{1,2}$ and $\kappa^{\text{intra}}_{1,2}$ because the direct coupling counts on the average permittivity but not the perturbation of asymmetric scatter, while the former is stronger than the later one. As a result, the lower branch is more lossy, making the upper branch favorable for lasing. Supplementary Fig. 7 illustrates how the system moves away from the EP by varying κ^{intra}_1, which represents the asymmetric coupling strength between the CW and CCW modes. Notably, the CW mode acquires a higher Q, rendering it more favorable for lasing.

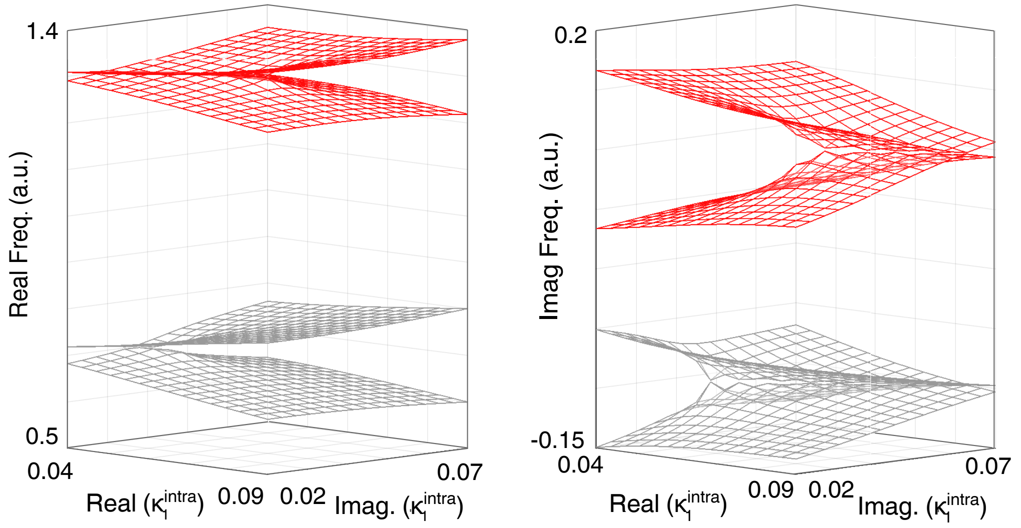

Supplementary Figure 7: **Complex bands as a varying coupling coefficient κ_1^{intra}.** The real part (left) and the imaginary part (right) of the eigenfrequency.

At the EP, the two eigenmodes in the upper branch turn out to be degenerate, corresponding to the same eigenvector of $\mathbf{V} = [0, 1, 0, 1]^T$. Therefore, they represent a collective mode that rotates in a CW direction in both the upper and lower layers. We define the degree of chirality (DoC) as

$$\alpha_{ch} = \frac{|a_{CW}|^2 - |a_{CCW}|^2}{|a_{CW}|^2 + |a_{CCW}|^2} \tag{S26}$$

as the contrast between the CW and CCW components, in which α_{ch} varies from $[-1, 1]$ with ± 1 representing the pure helicity in CW or CCW directions, respectively. Thus, the above solved state exhibits the maximum degree of chirality of 1. The condition of $\kappa_1^{\text{inter}} = -\kappa_1^{\text{intra}}$ indicates a perfect balance between the interlayer and intralayer coupling strength, which is difficult to control in experiments because of the complexity of asymmetric scattering. When κ_1^{intra} deviates from the ideal EP within the shaded region in Fig. 3b in the main text, the real parts of the eigenfrequencies remain almost degenerate. However, the difference in their imaginary parts

allows one mode to prevail in the mode competition, thus enabling single-mode lasing. As a supplement, we calculate the DoC near the EP region as shown in Supplementary Fig. 8, showing that the lasing emission can still be considerably chiral in the non-ideal case, corresponding to observable fork patterns through self-interferences.

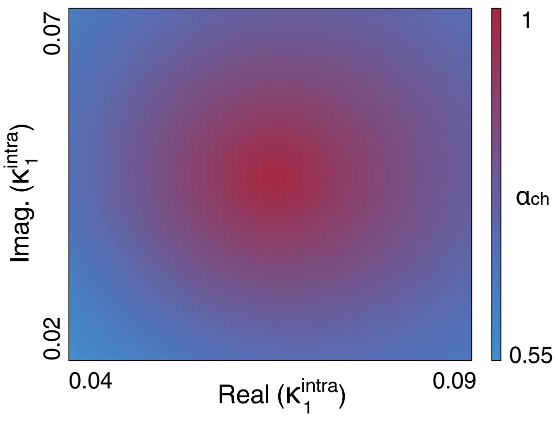

Supplementary Figure 8: **The chirality near the non-Hermitian degeneracy of EP as varying $\kappa_1^{\mathbf{intra}}$.** The degree of chirality (DoC) α_{ch} of the eigenmode.

Supplementary Note 7. Detailed measurement system for orbital chiral lasing

The detailed experimental setup is illustrated in Supplementary Fig. 9, featuring a confocal microscopy system that utilizes a free-space laser as the incident light source. To ensure optimal alignment, a height-adjustable mount is placed in front of the laser, enabling precise tuning of the beam along the optical axis of the setup. The laser employed is a pulsed source operating at 1064 nm (MPL-N-1064-200uJ), with a repetition rate of 10 kHz and a pulse duration of 2 ns. For illumination, a halogen lamp is used in conjunction with a lens L_4 ($f = 150, \mathrm{mm}$) positioned in front of it. This lens is confocal with the objective lens, providing accurate and stable illumination for sample alignment.

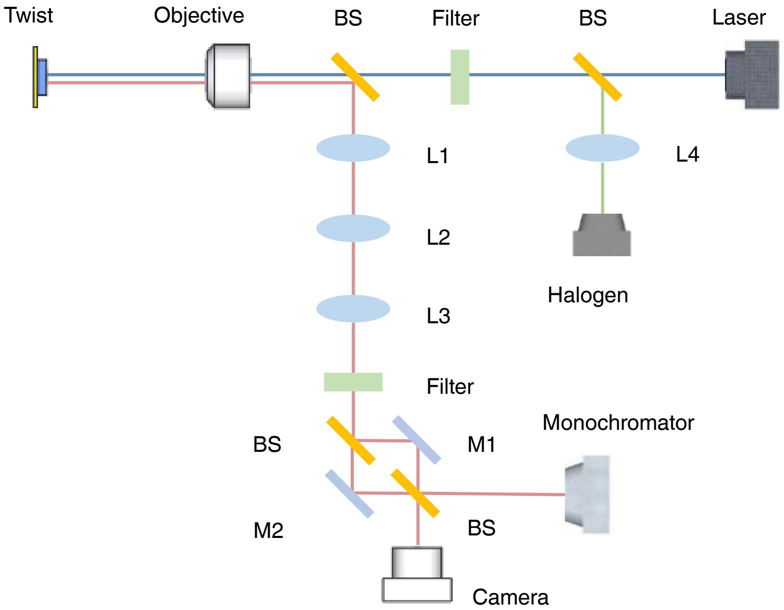

Supplementary Figure 9: **The detailed experiment setup.** The illumination is performed by using the Halogen light source. The Lens parameters are given in the Method section.

Supplementary Note 8. Characterization of the gain-induced effective cavity

As discussed, the lateral light confinement in our design is achieved through a gain-induced cavity. In this section, we present experimental evidence showing that the spatial localization of the lasing mode is governed by the pump beam profile.

First, we observed that the lasing mode consistently aligns with the position of the pump beam. To verify this, we hold the pump beam fixed while translating the sample by using a motorized stage. We ensured that the pump spot remained within the metasurface region throughout the measurement. Notably, the position of the lasing mode stayed stationary despite the movement of the sample. This behavior indicates that the lasing mode is confined by the gain-induced effective cavity created by the pump beam, rather than by the physical boundaries of the metasurfaces. Detailed results are provided in the Supplementary video.

Second, we varied the pump beam's size and found that the lasing beam's size changed accordingly. As shown in Supplementary Fig. 10a, we let the camera operate with fixed exposure time and gain settings to avoid automatic adjustments, which might interfere accurate measuring of beam sizes. We recorded the near-field profiles of the lasing mode after filtering out the pump light. As the pump beam expanded, the lasing mode also exhibited a corresponding increase in size. This trend was also evident in the far-field measurements. By removing lens L_2, we switched the system to a far-field configuration and observed that the far-field pattern became more focused (shrinking in angular spread) as the pump beam expanded. These results confirm that the size of the lasing mode is directly governed by the size of the incident pump beam.

Furthermore, we varied the pump energy while keeping its size and position fixed. Specif-

ically, we opt for incident pump powers of 3.5 μW, 8 μW, and 18 μW, and the corresponding mode profiles were recorded under identical camera exposure and gain settings. As shown in Supplementary Fig. 11, the lasing mode area increases slightly with higher pump power. This observation is consistent with the theoretical expectation that increased pumping enhances carrier diffusion, thereby enlarging the effective gain region available for lasing. Nevertheless, the expansion in mode size is relatively modest.

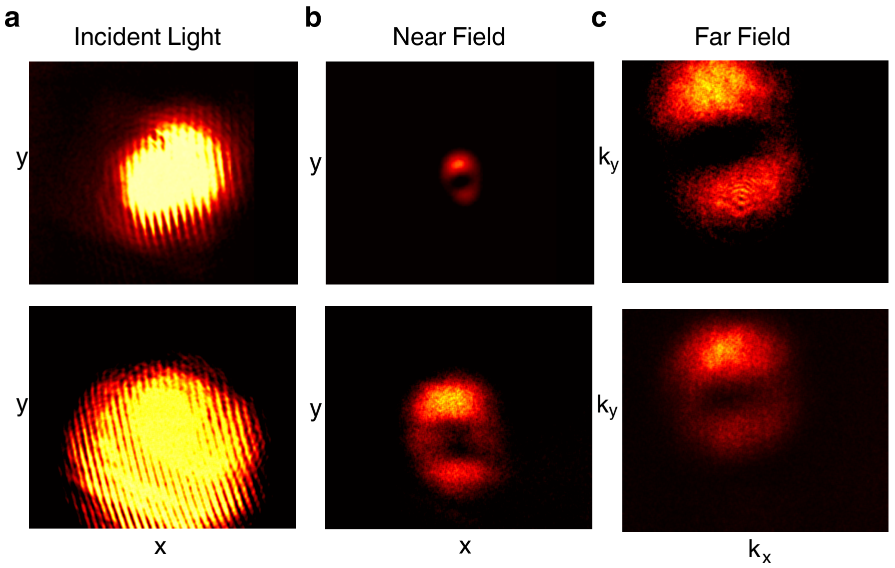

Supplementary Figure 10: **Effect of changing pump beam size on the lasing beam size.** (a) The pump beam with different sizes observed from the COMS camera. (b) The near-field images of the lasing beams at different pump sizes. (c) The far-field patterns of the lasing beam at different pump sizes.

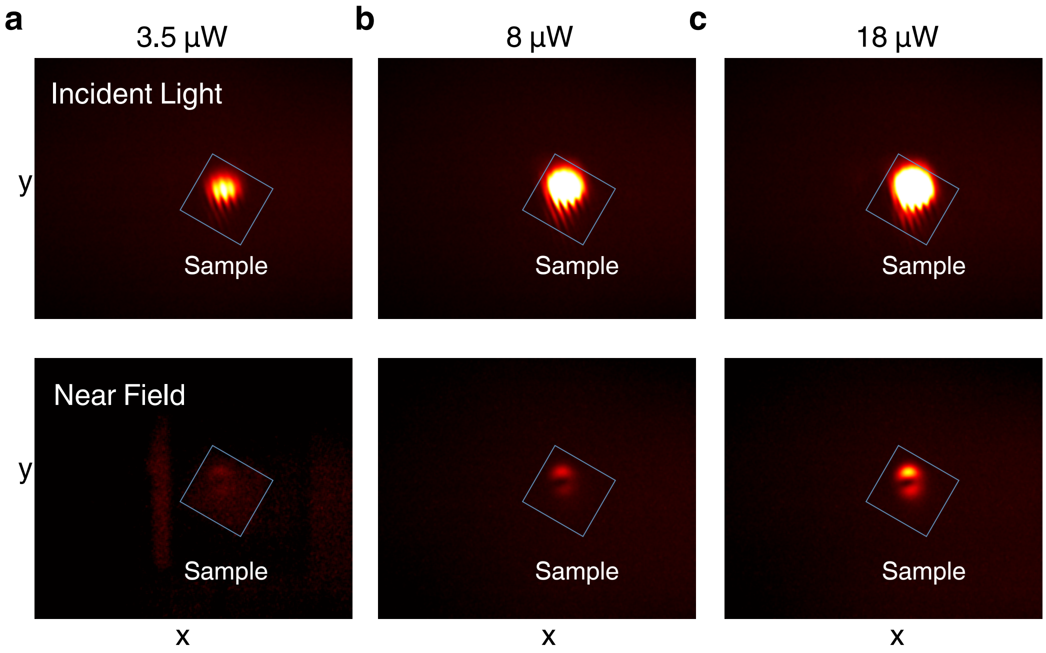

Supplementary Figure 11: **Effect of changing pump beam power on the lasing beam.** The upper and lower panels show the CMOS camera images of the pump beam and lasing beam in real space, respectively. The boxes visually indicate the physical boundary of the TBPhC sample. Different pump powers are applied for (a) 3.5 μW, (b) 8 μW, and (c) 18 μW.

Supplementary Note 9. The detailed observation of the lasing process

The experimental results presented in Fig. 5 in the main text reveal that our twisted metasurface operates in a single-mode oscillation regime. As a supplement, we provide additional data to further confirm the establishment process of single-mode lasing behavior.

We begin with a low pump power of 72.5 kW/cm^2 below the lasing threshold, with its spectrum shown in Supplementary Fig. 12a. At this stage, the amplified spontaneous emission (ASE) noise is prominent, nevertheless, two adjacent peaks can still be identified. These peaks correspond to the nearly degenerate eigenstates of the upper branch, as discussed in the previous section. The lower branch remains undetected due to its inherently lossy nature. This observation also suggests that our twisted metasurface system operates close to an EP condition.

As the pump power is increased to 72.8 kW/cm^2, both peaks become more pronounced and coexist (Supplementary Fig. 12b), indicating that the two modes are still competing for lasing. Upon further increasing the pump power to 74 kW/cm^2, the mode competition resolves, and a single mode dominates the spectrum, as shown in Supplementary Fig. 12c. This transition indicates that only the most favorable mode with a sufficient gain can surpass the lasing threshold to lase, while the competing mode is fully suppressed. During this lasing process, no external perturbation (such as asymmetric pumping we reported [13]) was applied, showing that the twisted metasurface system inherently supports mode-selection for chiral emission.

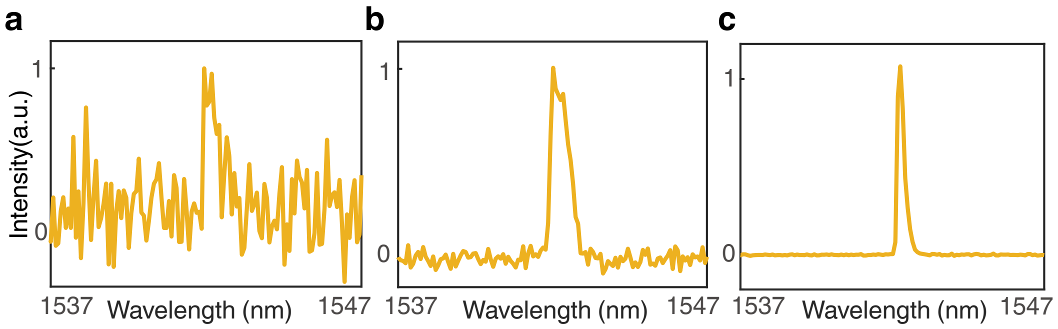

Supplementary Figure 12: **The detailed lasing process through increasing the pump power.** (a) Two almost degenerate modes appear on the ASE noise floor at the pump power of 72.5 kW/cm^2 which is below the lasing threhold. (b) The lasing oscillation is established at the pump power of 72.8 kW/cm^2, evidenced by significant suppression of ASE emission, but the two quasi-degenerate modes still exist. (c) The lasing transitions into single-mode lasing at the pump power of 74 kW/cm^2, only one clear lasing peak exists.

Supplementary Note 10. Comparison to the single-layer sample without intrinsic chirality

To verify the chirality of twisted bilayer metasurface intrinsically support orbital-chiral lasing, we fabricated single-layer reference samples that retain an achiral geometry. These samples share the same structural parameters, such as the air-hole period and radius, as the bilayer metasurface. The SEM images of the fabricated structures are shown in Supplementary Fig. 13 and a representative lasing spectrum is presented in Supplementary Fig. 14, showing almost one single peak across 20 nm wavelength range. A tiny peak appears adjacent to the main peak in the short-wavelength end, which may due to the residual weak chiral symmetry breaking due to fabrication imperfection.

Furthermore, we performed self-interference measurements on the single-layer samples. Because the excitation spot is smaller than the sample footprint, we performed measurements at two distinct excitation positions on each sample to ensure repeatability. To ensure that any potential fork features would not be overlooked, we expanded the field of view during imaging, the results are presented in Supplementary Fig. 15. Across all three samples and two excitation positions per sample, no fork patterns were observed. This confirms that the lasing modes in the single-layer structures are non-chiral.

For comparison, we measured two additional bilayer samples at two different excitation positions, as shown in Supplementary Fig. 16. In all cases, clear fork patterns were consistently observed. This confirms that the structural twist is indeed responsible for enabling orbital chiral lasing.

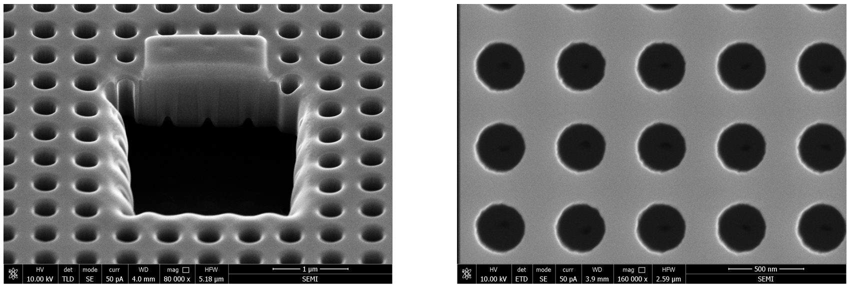

Supplementary Figure 13: **The SEM images of single-layer sample.** The over view of the metasurface with (left) cleaved side-walls and (right) the top view.

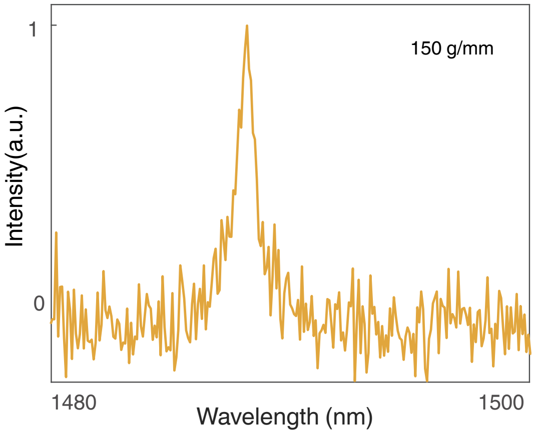

Supplementary Figure 14: **A presentative lasing spectrum of single-layer sample.** A nearly single peak is observed over a 20 nm wavelength range. A minor peak emerges adjacent to the main peak at the short-wavelength end, which may be attributed to residual weak chiral symmetry breaking induced by fabrication imperfections.

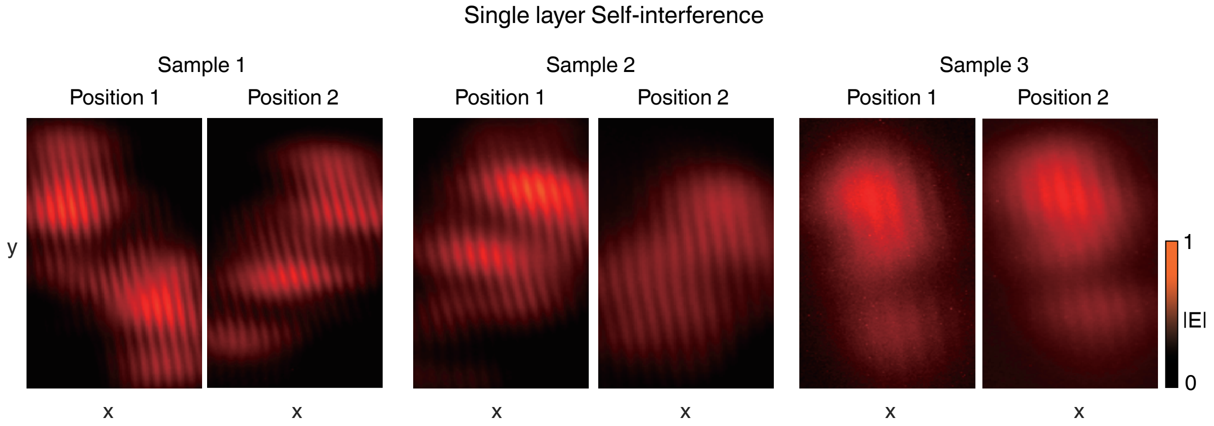

Supplementary Figure 15: **Self-interference of single-layer samples.** The interference patterns are presented for 3 samples each at 2 positions, no fork features were observed.

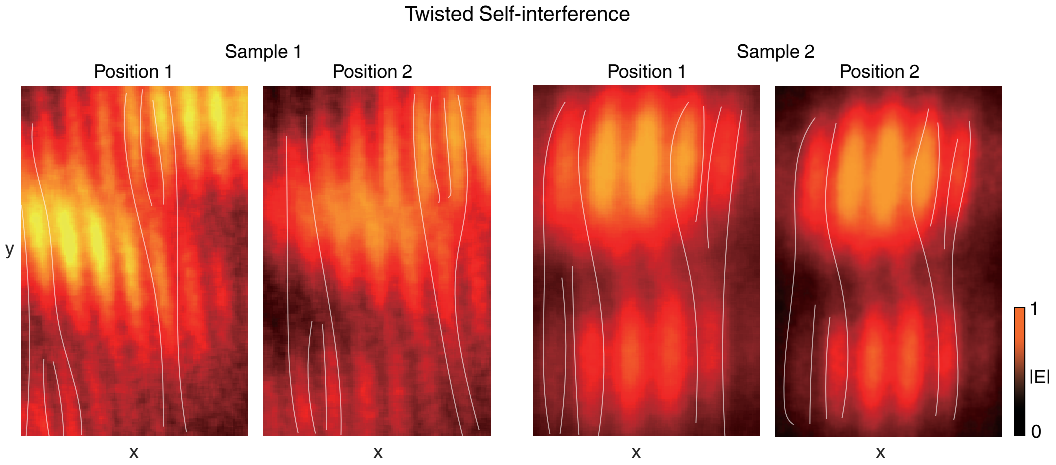

Supplementary Figure 16: **Self-interference of twisted bilayer samples.** The interference patterns are presented for 2 extra samples each at 2 positions, two-branch fork features were consistently observed with the same oritention.

Supplementary Note 11. Discussion on carrier dynamics

To clarify the observed vortex behavior belong to a steady state rather than transient response, we present a numerical model to analysis the carriers dynamics. In this work, we treat the gain medium as an isotropic profile in the hierachiral perturbation approach, thus the gain medium is approximated as achiral and linear for simplicity.

We consider the rate equation for the carrier density N inside the active region (InGaAsP MQWs), which is given by

$$\frac{dN}{dt} = \frac{\eta J}{qd_{\text{active}}} - (R_{\text{sp}} + R_{\text{nr}}) - \sum_{m} v_{gm} g_m N_{pm} \tag{S27}$$

where η is the optical efficiency of our laser, J is the current density, q is the elementary charge, R_{sp} is the spontaneous recombination rate, R_{nr} is the nonradiative recombination rate, v_g is the group velocity of the mode, g is the gain, N_p is photon density, and a separate photon density for each mode indexed by the interger m. For simulations, the temporal change curve of carrier density is calculated by solving equations:

$$\frac{dN_{pm}}{dt} = \left[\Gamma_m v_{g_m} g_m - \frac{1}{\tau_{pm}}\right] N_{pm} + \Gamma_m R'_{sp_m} \tag{S28}$$

For simplicity, in this study, we can approximate the gain spectrum by

$$g(N) = \frac{g_{\max} \cdot (N - N_{tr})}{N + \frac{g_{\max}}{|g_0|} \cdot N_{tr}} \tag{S29}$$

Supplementary Table 1: Parameters used for simulations.

Symbol	Parameter	Value	Source
J	Current density	2×10^7 A/m^2	–
d_{active}	Total thickness of Quantum wells	45 nm	–
τ_{sp}	Carrier lifetime	1.5 ns	–
Γ	Optical confinement factor	31.8%	Calculated by the TMM
n_g	Group refractive index	2.94	Calculated by the TMM
g_{max}	Maximum gain	1000 cm^{-1}	–
N_{tr}	Transparency carrier density	1.5×10^{24} m^{-3}	–
g_0	Absorption coefficient	−5000 cm^{-1}	–
α_{in}	Internal loss of materials	5 cm^{-1}	–
β	Spontaneous emission factor	1×10^{-4}	–

We plot the carrier dynamics of laser sample in Supplementary Fig. 17 based on the data in Supplementary Table 1. As can be seen from this curve, the carriers are in a steady state at 2 ns. Therefore, for a pulse duration of 2 ns, the system can be considered to operate in a steady state, without exhibiting any nontrivial temporal response.

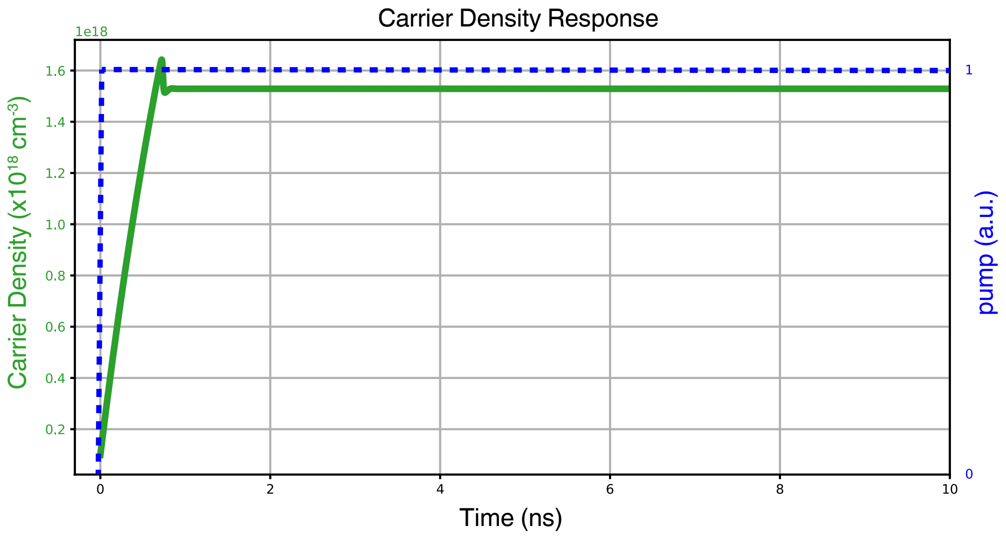

Supplementary Figure 17: **Carrier dynamics of laser sample.** The system reaches a steady state of carrier population before 2 ns, thus operating without exhibiting nontrivial temporal response for pulse durations matching or exceeding this timescale.

Supplementary Note 12. Discussion on the principles of OAM generation

To elucidate the specific merits of the twisted bilayer metasurface presented in this work, we categorize existing OAM generation principles into three primary classes: **local**, **non-local**, and **collective** mechanisms. These categories are distinguished by their interactions in real versus momentum space, and whether the OAM origin is intrinsic or extrinsic.

The local design principle relies on wave interactions in a tight-binding manner, widely implemented in single-layer metasurfaces or waveplates. In these structures, OAM is generated directly and locally through geometric variations, such as the introduction of Pancharatnam-Berry (PB) phases. While this approach offers substantial flexibility for tailoring wavefronts, the structures typically exhibit relatively low Q factors. Consequently, local designs are predominantly utilized for passive, extrinsic OAM generation rather than active lasing applications.

Non-local designs are typically realized in bulk photonic crystal (PhC) slabs, where modes exhibit long-range correlations. Wavefront manipulation in this regime occurs in momentum space, often associated with polarization singularities such as BICs. These systems can support sufficiently high Q factors, making them suitable for lasing. However, they generally function as extrinsic OAM generators, requiring specific external excitation conditions (e.g., circularly polarized pumping) to prepare the desired input state.

Collective designs represent a hybrid mechanism. From a local perspective, real-space coupling between sites gives rise to collective lattice resonances; from a non-local perspective, discrete guided resonances in momentum space hybridize into collective modes. This category allows for wavefront engineering in both real and momentum spaces. Examples include circular PhC cavities with collective guided resonances and PhCs with topological defects.

The twisted bilayer metasurface proposed in this work falls within this collective category but possesses a unique advantage: the OAM generation is **intrinsic**. Unlike extrinsic methods that depend on the pump profile, our system derives its chirality directly from the built-in structural twist. By leveraging exceptional points (EPs) and tailoring the modal Q factors, the system inherently distinguishes between clockwise (CW) and counterclockwise (CCW) rotating states. This mechanism enables active, single-mode OAM lasing that is robustly selected by the structural chirality itself, without the need for asymmetric pumping or external polarization control.

Supplementary References

[1] Ma, R.-M. *et al.* Twisted lattice nanocavity with theoretical quality factor exceeding 200 billion. *Fundam. Res.* **3**, 537–543, DOI: 10.1016/j.fmre.2022.11.004 (2023).

[2] Mao, X.-R., Shao, Z.-K., Luan, H.-Y., Wang, S.-L. & Ma, R.-M. Magic-angle lasers in nanostructured moiré superlattice. *Nat. Nanotechnol.* **16**, 1099–1105, DOI: 10.1038/s41565-021-00956-7 (2021).

[3] Luan, H.-Y., Ouyang, Y.-H., Zhao, Z.-W., Mao, W.-Z. & Ma, R.-M. Reconfigurable moiré nanolaser arrays with phase synchronization. *Nature* **624**, 282–288, DOI: 10.1038/s41586-023-06789-9 (2023).

[4] Luo, X.-W. & Zhang, C. Spin-twisted optical lattices: Tunable flat bands and larkin-ovchinnikov superfluids. *Phys. Rev. Lett.* **126**, 103201, DOI: 10.1103/PhysRevLett.126.103201 (2021).

[5] Dong, K. *et al.* Flat bands in magic-angle bilayer photonic crystals at small twists. *Phys. Rev. Lett.* **126**, 223601, DOI: 10.1103/PhysRevLett.126.223601 (2021).

[6] Wang, H., Ma, S., Zhang, S. & Lei, D. Intrinsic superflat bands in general twisted bilayer systems. *Light Sci. Appl.* **11**, 159, DOI: 10.1038/s41377-022-00838-0 (2022).

[7] Yi, C.-H., Park, H. C. & Park, M. J. Strong interlayer coupling and stable topological flat bands in twisted bilayer photonic moiré superlattices. *Light Sci. Appl.* **11**, 289, DOI: 10.1038/s41377-022-00977-4 (2022).

[8] Huang, L., Zhang, W. & Zhang, X. Moiré quasibound states in the continuum. *Phys. Rev. Lett.* **128**, 253901, DOI: 10.1103/PhysRevLett.128.253901 (2022).

[9] Siegman, A. E. *Lasers* (University Science Books, 1986).

[10] Forbes, A., Mkhumbuza, L. & Feng, L. Orbital angular momentum lasers. *Nat. Rev. Phys.* **6**, 352–364, DOI: 10.1038/s42254-024-00715-2 (2024).

[11] Kawase, D., Miyamoto, Y., Takeda, M., Sasaki, K. & Takeuchi, S. Observing quantum correlation of photons in laguerre-gauss modes using the gouy phase. *Phys. Rev. Lett.* **101**, 050501, DOI: 10.1103/PhysRevLett.101.050501 (2008).

[12] Zhang, T. *et al.* Twisted moiré photonic crystal enabled optical vortex generation through bound states in the continuum. *Nat. Commun.* **14**, 6014, DOI: 10.1038/s41467-023-41068-1 (2023).

[13] Chen, Y. *et al.* Observation of chiral emission enabled by collective guided resonances. *Nature Nanotechnology* **20**, 1205–1212, DOI: 10.1038/s41565-025-01964-7 (2025).
