# Supplementary material for: Chiral orbital lasing in a twisted bilayer metasurface
Source: Nat Commun. 2026 Mar 12;17:2369. doi: 10.1038/s41467-026-69665-w (PMC12982590; doi:10.1038/s41467-026-69665-w)
Supplement: Supplementary file 2 — Description of Additional Supplementary Files [file 41467_2026_69665_MOESM2_ESM.pdf]

Description of Additional Supplementary Files

File Name: Supplementary Movie 1
Description: **The position of the lasing mode stayed stationary despite the movement of the sample.** The lasing beam follows the pump beam to its new location while maintaining its doughnut shape, confirming the effectiveness of the gain-guiding mechanism.
